# Tools for public health policy: nudges and boosts as active support of the law in special situations such as the COVID-19 pandemic

**DOI:** 10.1186/s12992-021-00782-5

**Published:** 2021-11-20

**Authors:** Jakub M. Krawiec, Olga M. Piaskowska, Piotr F. Piesiewicz, Wojciech Białaszek

**Affiliations:** 1 SWPS University of Social Sciences and Humanities, Institute of Psychology, DecisionLab: Center for Behavioral Research in Decision Making, Warsaw, Poland; 2 SWPS University of Social Sciences and Humanities, Institute of Law, Warsaw, Poland

**Keywords:** Boosts, Nudges, Law, COVID-19

## Abstract

In recent years, “nudging” has become a standard behavioral intervention at the individual level and for the design of social policies. Although nudges are effective, such interventions seem to be limited to a given space and time, and there is only scant evidence to support the contrary view. On the other hand, choice architects may utilize another type of intervention called “boosting,” which shows the promise of generalized and lasting behavioral change. A government can use these tools to shape public policy. Behavioral interventions such as policy-making tools have their boundaries, as does the law. We argue that nudging and boosting may serve as active local or global aids in support of the legal system under certain circumstances. Nudging and boosting can also support the legal system, especially in relation to emerging social issues or events that are unprecedented, such as the recent global COVID-19 pandemic, where certain behavioral patterns are expected, but it would be difficult or impossible to enforce them through the law alone.

## Introduction

On the 9th of November, Pfizer was the first company in the world to announce the success of its vaccine, which was approximately 95% effective in producing immunity against the novel coronavirus [1]. Thus far, the global pandemic has directly affected more than 224 million individuals, leading to 4.63 million casualties [2]. Whether the vaccine is available to all citizens or not, one effective way of taking back control over the increase of infections is to follow the simple rules of social distancing, wearing masks, and disinfection, supported by the reliable education of society. These measures were widely adopted as official means of disease prevention recommended by the World Health Organization [3]. Despite these recommendations, which were generally imposed by governments in the form of temporary measures, some individuals have not been complying with these new public health measures [4, 5]. The prescriptive (how one should behave) legal system might be vulnerable if individuals tend to ignore new restrictions, leading to the inefficiency of new laws [6]. Legislation and laws have their boundaries, and behavioral interventions such as nudges and boosts may serve as active aids in support of such prescriptive systems, as discussed in the following sections.

In recent years, governments and public officials have shown a growing interest in using the behavioral sciences to promote policy goals. An example is an Executive Order issued by the US President Barack Obama, which recommended federal agencies to use behavioral science to shape public policies [7]. In Europe, a similar approach has been proposed by the Behavioral Insights Team [8]. Such strategies assume that the use of tools from the behavioral sciences can guide human behavior and achieve public policy objectives. Apart from the effectiveness of the behavioral sciences in shaping an individual’s choice, the question is whether and to what extent it is possible for the state to use behavioral intervention tools.

The main aim of the current paper is to show how the law can be supported by behavioral interventions. Although previous research has proposed the use of the law in combination with one behavioral intervention – i.e., “nudging” [9], we further propose the use of “boosting” to achieve governmental and individual goals. In our view, nudges and boosts are an integral part of (and not a substitute for) the formal regulations of modern societies, where freedom of choice and autonomy should be promoted concurrently in line with public goals. In further sections, we accomplish our main aim by introducing and defining nudges and boosts, outlining classical law-based approaches to behavioral change, showing examples of the boundary areas in which additional enhancement is needed, and providing tools (i.e., nudges and boosts) by which behavioral change can be accomplished. Furthermore, we provide extended taxonomic information about nudges and boosts, followed by examples of the most popular interventions used so far in such domains as finances, the environment, and health. We argue that the legal system and behavioral interventions do not exist in a vacuum. Attempts to understand the boundaries of the legal system and to treat behavioral interventions as active support for the law may yield stronger policies and thus provide real change at the societal or individual level. To our knowledge, this is the first attempt to collate a coherent theoretical framework, with examples of behavioral interventions used during the COVID-19 pandemic, which stems directly from the presented taxonomies.

### Tools for behavioral change – nudges and boosts

One of the most widely used approaches for behavioral change at the individual and societal levels is nudging. The definition of “nudge” has several different forms in the literature, with the variation being generally in the number of categories of interventions included in the nudging, the function of the behavioral interventions, and how the nudges influence the architecture of the decision-making, as well as the decision-makers themselves [10]. However, the main concepts entailed in the nudge approach are based on the definition proposed by Thaler and Sunstein [11], who define the concept of nudge as “any aspect of the choice situation architecture that predictably changes behavior, without prohibiting any options, or significantly changing economic benefits.” The introduction of nudging has prompted intense discussion on the ethics, hopes, and potential applications of the concept of nudging in social policy [10]. Due to its generally low implementation costs, the nudge approach, which by definition does not take into account financial incentives or penalties, has become intensively developed, especially in the domains of financial, health, and environmental decisions [12].

Although nudging has been promoted as an effective means of changing human behavior through interference with the architecture of the decision-making situation [13], it is not the only approach that can be used to change behavior effectively. The second method is more recent but no less promising, namely the “boost” approach proposed by Grüne-Yanoff and Hertwig [14]. While the main impact of a nudge relates to the architecture of the decision-making situation, which consequently influences decision-making behavior, the main aim of a boost is to increase the competence of the decision-maker to make the best possible decisions, according to the decision-maker’s preferences and whatever is appropriate in a given situation [15]. Despite the fact that nudging and boosting operate in similar areas concerning choice architecture, supporters of the boost approach distinguish several differences between them. Most importantly, they postulate that nudging in contrast with boosting: (1) can have a potential impact on the autonomy of the decision-maker; (2) can have possibly reversible effects when the intervention is withdrawn; and (3) uses cognitive biases as the basis for its impact. Furthermore, the proponents of boosts claim that boosts, produce long-term effects and affect the decision-maker’s competence [15]. Although the proponents of the boosting approach claim that boosts do not disrupt decision-makers’ autonomy, in our opinion this statement is not entirely true. When boosts are chosen on a subjective, individual level, freedom of choice is maintained; however, when political or ideological reasons are used to advocate boosts, they may limit the autonomy of choice, just as nudges do. Although this type of intervention looks more neutral, when a boost is proposed by a higher authority, a certain style of action in a given situation may be imposed.

### Taxonomies and popular examples of nudges and boosts

The taxonomies of nudges and boosts, which we present in this section, are derived from the work of Sunstein [16] and Hertwig and Grüne-Yanoff [15]. The taxonomy of nudges presented below originally consisted of ten different interventions [16]. In their meta-analysis, Hummel and Maedche [17] went further in proposing two categories of interventions, noting that while theirs may not be the complete list of nudge types, it appears to be the most exhaustive [17]. In other words, the taxonomy presented in Table 1 assumes that choice architects in different contexts may wish to nudge people by presenting them with a chosen, limited set of particular options, or by presenting the options in a particular way. The category of nudges called *structuring the choice task* refers to what to present, whereas the category called *describing choice options* represents how choice options are presented. We decided to exclude a tool called *implementation intentions* from the second category because this paper generally assumes that this tool is a boost that can be taught to individuals, rather than being an automatic nudge that is incorporated into a setting. Implementation intentions was a strategy developed originally by Gollwitzer [18], which links imagined situations with prepared responses; for instance, “Whenever situation x occurs, I will initiate response y.” The effective use of this tool demands individually conscious planning, which is a skill more likely to be applied by someone with a good awareness of the topic. Following Hertwig and Grüne-Yanoff [15], we have included this in the boost taxonomy.

**Table 1:** Taxonomy of nudges showing examples of nudges referring to structuring the choice task and those describing choice options

Nudges structuring the choice task	Nudges describing choice options
1. Default	1. Warnings/graphics
2. Simplification	2. Reminders
3. Social reference	3. Precommitment
4. Change effort	4. Feedback
5. Disclosure	

This taxonomy is based on Sunstein [16], and further developed by Hummel & Maedche [17]

The taxonomy of boosts (Table 2) focuses on three categories: *risk literacy boosts* (risk and probability knowledge), *uncertainty management boosts*, and *motivational boosts* [15]. These interventions are divided into separate categories in order to emphasize their vital aims: *risk literacy boosts* are targeted at developing competences to help individuals better understand statistical information, *uncertainty management boosts* establish step-by-step rules to make good decisions or predictions, and *motivational boosts* aim to develop a person’s ability individually to fine-tune his/her own motivation, cognitive control, and self-control, in an overall sense. Furthermore, there is a new tool in the boost taxonomy that was not included by Hertwig and Grüne-Yanoff [15]. This is called “inoculation strategy,” which was identified as a boost by Kozyreva et al. [19]. Inoculation refers to the process of giving a psychological vaccine to individuals by training them in the process of recognizing particular information [20]. It was assigned to the category of “Uncertainty management boosts” in this taxonomy due to its potential for reducing uncertainty [21]. The two taxonomies of nudges and boosts presented in Tables 1 and 2 appear to be the most exhaustive categories, taking into account the most prominent research concerning ways of constituting choice architecture.

**Table 2:** Taxonomy of boosts showing examples of boosts referring to three categories: risk literacy boosts, uncertainty management boosts, and motivational boosts

Risk literacy boosts	Uncertainty management boosts	Motivational boosts
1. Graphical representations	1. Simple actuarial inferential methods	1. Expressive writing
2. Experienced-based representations	2. Simple rules of collective intelligence	2. Growth-mindset or sense-of-purpose exercises
3. Representations avoiding biasing framing effects	3. Fast and frugal decision trees, simple heuristics, & procedural routines	3. Attention and attention-state training
4. Brief training in transforming opaque representations 5. Training of general math skills	4. Inoculation strategies	4. Psychological connectedness training 5. Reward-bundling exercises 6. Implementation intentions & other use of automatic processes 7. Precommitment strategies 8. Self-control strategies

This taxonomy is based on Hertwig & Grüne-Yanoff [15], and further developed as suggested by Kozyreva et al. [19]

### The legal system and its boundaries

Inspection of the relationship between the state and the law is fundamental to considering whether the state can use behavioral tools, such as nudges and boosts, and whether the state can choose its tools freely. First, the law determines the framework for the functioning of the state, including the areas of permitted interference by authority. The law defines the systemic framework for the functioning of authorities and determines their tasks. The relationships between the state and the law are connected to the functions of the state. Second, the law is the basic tool of state activity, and in this sense it is related to the internal functioning of the state. The external functioning includes the activity of the state in the area of international relations, which mainly involves the protection of its interests and issues of external security, such as the development of international relations (e.g., international politics, economy, and culture), whereas the internal functioning involves the relationships between the state and society and the individual within the structure of a specific state. Therefore, internal functioning is related to the issues of ensuring public safety and order, the protection of property, securing durability in the internal structure of social relations, and the protection of health and the environment. Here, the state regulates the activities of individuals, focusing on behaviors that are desirable or undesirable from the point of view of implementing a specific task for which the state was established. Thus, by using tools that are within the state’s authority, the state can influence the behavior of an individual, including via the use of specific coercion. This leads to the adoption of an attitude considered by the state to be socially desirable. This internal domain concerns issues regarding the relationship between individual interests in which the state does not interfere, and above all, the public interest.

The issue of the limits of the legal system emerges in the area of the internal function of the state. When serving this function, the law is understood as an instrument for exercising the power of the state. The question about the limits of the law in this sense is, de facto, a question of how far the state can intervene using this tool. This is a universal question that individual states and state authorities have considered for many years, and lies beyond the scope of this article. However, it is important to attempt to delineate those boundaries for which the relationships between public and private interests are important. This relationship is closely related to the system adopted by a given state. There is no doubt that public and private interests in a totalitarian or authoritarian state will have meanings other than those in a democratic state ruled by law. Thus, depending on the political system, the rules defining the object of state power, the directions of state activity (related to the public interest), and issues related to civil rights and freedoms (private interest) will be different.

A state’s political system determines the areas of its activity that can be implemented by it within its framework, based on applicable law. The scope of possible interference of the state’s authority is, in other words, the permissible limits of the operation of the law as the basic tool used by the state, as adopted by it under a specific system. At the same time, it seems reasonable to say that these boundaries are now becoming narrower, because states also use the tools of behavioral intervention, doubtless because in some cases they appear to be effective.

Apart from the law, which is still the traditional tool for the exercise of state power, there are other tools that sometimes replace it. Therefore, the question arises of whether – since we can talk about the boundaries of the law understood as a limit of the activity of state power established within a specific system, or leaving a specific sphere free from regulation – we can talk about the boundaries of using other tools that are not traditional (including behavioral interventions). It seems that the answer lies in the relationship between public and private interests, and more specifically, in determining interference with civil rights and freedoms. This issue is related to the implementation of the internal functions of the state, and therefore, solving problems that arise with regard to public policies that should be implemented based on and within the law.^1^

To ensure the effectiveness of actions taken as part of public policies, the state uses specific tools that can be combined. In this sense, the law, as the basic tool of the state for shaping public policy, can be supported by other tools aimed at shaping specific behaviors by individuals (nudging or boosting).

### Traditional approaches to the public health system, recommendations, and legal instruments

One of the areas that falls within the scope of public policy concerns is health policy, as related to public health in particular. We can define public health as: “an empiric and multidisciplinary field whose goal is to assure conditions in which people can be healthy” [23]. The literature on the subject indicates that health policy has both practical and scientific dimensions [24]. The WHO defines health policy as: “health goals at the international, national, or local level, and specifies the decisions, plans, and actions to be undertaken to achieve these goals” [25]. The WHO also states that “public health policies are developed through highly complex processes that involve different levels of government, and numerous stakeholders with diverse needs and interests” [25]. Authorities achieve health policy objectives through health-system governance, which, according to the WHO definition, should be understood as “a wide range of steering and rule-making related functions carried out by governments/decisions-makers, as they seek to achieve national health policy objectives that are conducive to universal health coverage” [26]. The concept of health-system governance in health policy also refers to “the processes, structures and organizational traditions that determine how power is exercised, how stakeholders have their say, how decisions are taken and how decision-makers are held to account” [27, 28]. It is up to the authorities of particular countries to define the objectives of this policy; hence, policy is generally implemented at the national level. Implementation of public health policy takes place, among others things, through the organization of prevention programs, the financing of public healthcare, and the financing or co-financing of specific medicines or medical devices. As a rule, health policy goals are long-term and are implemented over time. One important practical aspect of health policy lies in teaching members of society to be responsible for their own behaviors that affect health [29]. To achieve this goal, the state can use various tools to change people’s behavior.

The importance of health policy and the role of the state in shaping it have gained new meaning in the face of the COVID-19 pandemic. Action to prevent the spread of the virus is not just part of the domestic policy of a particular country, because the pandemic has affected the whole world. Therefore, the actions undertaken have also been coordinated at the international level. The WHO developed recommendations to combat the pandemic, dividing the required tasks into sixteen areas [3]. All these recommendations were aimed at countries in order to help fight the pandemic. The implementation of these solutions, as well as the search for new ways of fighting the pandemic, took place traditionally, i.e., through the introduction of appropriate legislative solutions. In the fight against the virus, the law became the primary tool used by state authorities.

Several of the restrictions aimed at preventing the spread of the virus pandemic were introduced at the national level in almost all the countries affected by the pandemic through the introduction of orders and bans that included the threat of sanctions. For example, there was an order to cover the mouth and nose, and obligatory quarantine for those who had been in contact with an infected person. However, temporary limitations introduced through legal acts are not always an effective way to fight a pandemic. The restrictions affect individual rights and freedoms, which the state had not previously considered. There is no doubt that laws are fast and efficient tools for shaping public policy and influencing human behavior. Yet, in the face of an increasing number of restrictions, many individuals refuse to comply with them, and as a consequence, these tools become less effective. In Poland, an example of such behavior was seen in the recent protests by women and other social groups (related to the ruling of the Constitutional Court regarding eugenic abortion) that took place despite the current ban on gatherings of more than five people. However, apart from the issues related to the observance of the law, we need to examine the limits of the permissibility of the state’s interference in the rights and freedoms of individuals in order to implement the overriding interest of public health. Moreover, if the traditional tool for shaping public policy is ineffective, should the state turn to behavioral interventions as tools? And what are the possible conditions for their use?

### Areas for enhancement through the application of behavioral tools

When discussing the boundaries of the law and the possible boundaries of the state’s use of other tools (i.e., nudges and boosts), the conditions for their use, and their scope and boundaries, we must first consider the areas of state activity (its functions) and second, the relationship between public and private interests.

#### Boundaries of the law depend on the areas of state activity

**Fig. 1:**
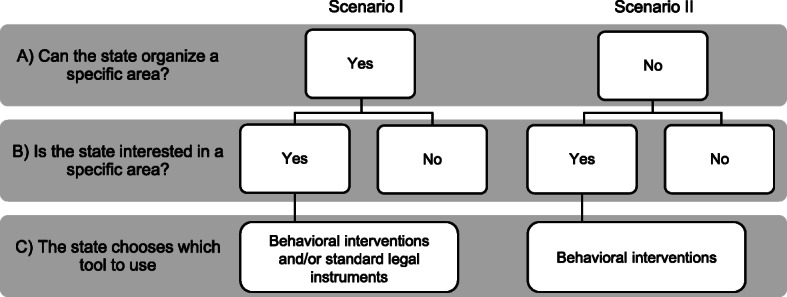
Decision flowchart showing the conditions in which the state may choose behavioral interventions and/or standard legal instruments to change behavior at the individual or societal level

We propose a theoretical framework to depict possible paths the state can follow when deciding whether to use behavioral interventions either alone or in combination with classical legal instruments (Fig. 1). Using the criteria of state functions and tasks performed by the state, on the first level (Fig. 1, Row A) we distinguish two main scenarios differing in whether: (1) the particular area is subject to the authority of the state, and (2) the area is not subject to this authority. The first includes the area of social and individual life, which from the point of view of the functions (both internal and external) of the state, belong to it, and the state can and should regulate them because they fall within the tasks entrusted to it. As a rule, the state’s activities are undertaken in pursuit of the public interest (the interest of society), and public interest prevails over private interest. Where the area is not subject to the state’s authority, there is a sphere that is beyond the scope of the state to regulate. There may be various reasons for this. First, because the autonomy of the individual is left to the individual, there are rights and civil liberties granted to him/her that the state should not violate. This is related to the specific system in force in a given country. For this reason, depending on the adopted state system, the area of the lack of authority over the life of an individual due to the protection of his/her rights may vary between countries. Second, the state is not interested in certain actions or activities of individuals and even of society as a whole. In simplified terms, some of this sphere can therefore be assigned to activities falling within private interests, which in this case may outweigh the public interest.

Depending on the system adopted in a given country, and on the specific objectives of public policies, individual areas and the problems solved within them may vary. In both of these aspects, however, it is possible to make a further division into those issues in which the state has an interest and those in which it does not (Fig. 1, Row B).

Scenario I Row A (see Fig. 1) is one in which the state can regulate a specific sphere of social life or individuals. However, not every issue that falls within this sphere will be subject to such regulation, because the state may not deem it expedient in every case to undertake actions to exercise its authority in cases that fall within this sphere. This lack of regulation may be justified, for example, by a practice that already exists in society, which realizes the goal set by the state. Thus, this makes it unnecessary to take imperative action: i.e., there is no need to regulate something that is already functioning efficiently and achieving an intended purpose. However, if a state is interested in regulating a specific sphere, it chooses the tool that will be used to achieve the assumed goal. The traditional tool used by the state is the law, although as already indicated, it is not the only tool that the state can use. From the foregoing, the following division follows at Row B: (1) an area where the state can regulate activity in which it is interested; and (2) an area where the state can regulate activity, but it is not interested in so doing.

When considering the main topic, which is the use of the law, nudges, or boosts, the area of interest is that where the state can impose regulations and is interested in doing so. A “no-visitor policy” in hospitals is a useful example of a public policy in the field of health. The health policy is part of the state’s activity and, as a rule, when taking action in this area, the state may use the tool of the law. In this situation, the public interest is the health of society as a whole, and the private interest is both the health of individuals and the rights and freedoms granted to them, in which the state should not interfere. A no-visitor policy serves a health-protection purpose for patients, staff, and potential visitors. Thus, it serves to protect the health of society as a whole, and to protect the health of individuals at the same time. The duty to ensure the efficient functioning of health services belongs to the state, and therefore, it may enter this area and establish such a prohibition. In this case, the state uses the tool of the law.

In Scenario II (Fig. 1), where the state cannot intrude upon social life or the life of individuals, the implementation of tools depends strongly on the state’s interest. There are various reasons for the state’s inability to regulate a specific sphere of an individual’s life, which are based mainly on the model of the system adopted in a given country, including the rights and freedoms of citizens, but not only on this. There are types of activities in which the state is not interested at all, e.g., whether a certain individual wears gloves in winter or not. Within this scenario, however, there are spheres of individual activity in which the state does have an interest because the specific behaviors of an individual serve society or help to achieve the goals of a specific policy. In this sense, it can be said that although the state has “agreed” not to enter a given sphere, it is interested in the activities undertaken within its framework, because these are related to the implementation of specific tasks of the state. From the foregoing, at Row B the following divisions follow: (1) an area that the state cannot regulate in which the state is interested; and (2) an area that the state cannot regulate in which the state is not interested.

An example of health policy during the COVID-19 pandemic is illustrated by donations of blood plasma. Introduction of a legal obligation to donate blood would violate the rights and freedoms of an individual granted by a legal act of the highest order. In such a situation, the state cannot create such an obligation. However, it is in the interest of the state that as many people as possible donate blood plasma, because this addresses the objective of making the fight against the pandemic more efficient, and thus influences the implementation of the health policy goals of the state. However, the state is interested in the attitudes of citizens towards donating blood. The inability to exert authority over some of an individual’s activities in this sphere using a traditional tool, which is the law, therefore raises the possibility of using the tools of behavioral intervention and the conditions for their use. This issue also falls within the framework of the considerations undertaken.

There may be some exceptions that are not illustrated in Fig. 1. The state may foresee extraordinary situations in which civil rights and freedoms may be limited to a greater degree than the standard adopted in the act of the highest order. In such a case, the rules and conditions under which the restriction of rights and freedoms may occur (e.g., the state of emergency mentioned in the Polish Constitution) are defined. In these exceptional cases, the state will also be able to regulate the area, which as a rule, lies outside the scope of its authority. The possibility of introducing limitations on the rights and freedoms of an individual may only take place exceptionally and under strict conditions. In this exceptional case, the sphere that as a rule falls outside the scope of the state’s regulatory authority is included in the scope of this authority.

The divisions presented in Fig. 1 indicate the boundaries of the law, i.e., the state’s use of a tool, such as the law. The state can only use “the law” if it operates in an area it can regulate (both in terms of the principle and the exception). In turn, the boundaries of the state’s use of nudges and boosts will depend on whether or not the state is interested in a specific area of individual activity.

#### Boundaries of the law depend on the relationship between public and private interests

The second boundary comes from the relationship between public and private interests. It is their mutual relationship that determines the scope of state interference through the use of a specific tool for shaping the behavior of an individual. In turn, the relationship between public and private interests should be linked to a specific system in a given country. These relationships are therefore different from the priority of the public interest over the private interest (to a different degree), and there is a certain balance between them. It seems appropriate to illustrate these connections using the example of a democratic state ruled by law, where a certain balance is assumed between these two interests. In this system, private interest is manifested, inter alia, in several rights and freedoms granted to citizens in a legal act of the highest order, which may be limited in this system only in exceptional cases. As a rule, the state does not enter this sphere.

The public interest is seen in a general clause, reference to which can be found in almost every legal system. Depending on the legal context in which this clause is applied by the legislator, it allows the authorities applying the law to refer to values that are common and important for the whole society, and in particular, are based on the values indicated in legal acts on which a specific system is based in the law (in Poland it is the Constitution). In a specific situation, the public interest may justify the limitation of human and civil rights and freedoms because the benefit to society as a whole from a certain state objective is of greater value than the private interest. Private interest, on the other hand, is the interest of the individual, and the relationship between an objective state and the assessment of this state from the point of view of the benefit that it brings or may bring to the individual [30].

It has been noted that two areas remain within our scope of interest. The first is the area that the state can regulate and is interested in. The second is the area the state cannot regulate and is interested in. In both cases, it is necessary to choose the tool that the state can use to achieve the assumed goal. There is no doubt that in the first case there is a choice between the law (the traditional tool), nudges, and boosts. In the latter, the choice is limited only to the tools of behavioral intervention, because this is an area that, in principle, the state cannot regulate. It thus remains to be considered what criteria the state should follow when selecting a specific tool, and after making a decision, and the selection of a specific solution. In this case, the relationship between public and private interests is also important.

### The proportionality principle

The choice of a specific tool should respect the principle of proportionality, which is applied when the use of a specific tool would limit or violate the rights and freedoms guaranteed by the state. Both the European Convention on Human Rights and the Charter of Fundamental Rights of the European Union (EU) provide for the possibility of restricting a specific right or freedom to protect the public interest in the broad sense [31, 32]. The meaning of public interest, as developed in the case law of the EU Court of Justice, includes, inter alia public policy, public security, public safety, public health, preserving the financial equilibrium of the social security system, the protection of consumers, recipients of services, and workers, the fairness of trade transactions, combating fraud, the protection of the environment and the urban environment, the health of animals, intellectual property, the conservation of the national historic and artistic heritage, social policy objectives, and cultural policy objectives [33]. In compliance with EU law, the proportionality principle is in the public interest [34]. Additionally, Article 52, Section 1 of the Charter of Fundamental Rights of the European Union [35] defines the criteria that must be applied for a specific regulation that limits rights and freedoms to meet the requirements of the principle of proportionality. According to that Article, any limitation on the exercise of the rights and freedoms recognized by this EU Charter must be provided for by law and must respect the essence of those rights and freedoms. Subject to the principle of proportionality, limitations may be made only if they are necessary and genuinely meet the objectives of general interests recognized by the Union or the need to protect the rights and freedoms of others. This provision clearly shows that when analyzing the proportionality of a tool used by the state, it is necessary to examine whether this tool meets each of the three auxiliary criteria, i.e., suitability, necessity, and proportionality sensu stricto.

When examining whether the criterion of suitability is met, it is necessary to examine the relationship between the purpose, which the introduction of a specific regulation serves, and the measures applied by the state. A measure is suitable when it is capable of achieving its intended purpose, which was previously determined by the state [36].

Investigating whether the established restrictions within the scope of exercising certain freedoms meet the criterion of necessity requires an answer to the question of whether “the same goal could not be achieved by using other means, less burdensome for the citizen, because they interfere less with his rights and freedoms” [37, 38].

On the other hand, the examination of whether the applied tool will meet the proportionality criterion in the strictest sense comes down to a balance between the assessed values. When applying these criteria, it is necessary to examine the relationship between the usefulness of the applied legal measure for the general good of a given Member State and the limitations experienced by treaty freedoms [39, 40]. The application and evaluation of tools, such as nudges and boosts, must be viewed through the prism of Article 52, Section 1 of the EU Charter of Fundamental Rights, which enables the assessment of their admissibility from a legal perspective.

### Comparison of behavioral interventions and legal instruments

As we have outlined, legal instruments have boundaries to their application, and their use depends on the state’s authority in a given area and the state’s interest in it. However, evaluation of the application of nudges, boosts, and legal instruments should not be guided solely from the state’s perspective. In this section, we compare three types of tools that help change behavior across several dimensions, which may provide further reasons for selecting each instrument (see Table 3). A more detailed comparison that focuses only on nudges and boosts can be found in Hertwig and Grüne-Yanoff [15].

**Table 3:** Dimensions distinguishing nudges, boosts, and legal instruments

Dimension	Nudges	Boosts	Law
Usual form	Stimuli implemented in decision situation	Acquisition of competencies	Regulations or prescriptions
Intervention target audience	Populations or individuals	Populations or individuals	Populations and groups
Implementing entity	Private, governmental or non-governmental organizations	Private, governmental or non-governmental organizations	Legislature (authorized body)
Costs of implementation	Low	Moderate to high	High
Individual level costs to follow	Low	Moderate to high	Moderate to high
Implementation time	Short	Moderate	High
Duration of action	Effective during intervention	Resulting competences may exist long after the intervention ends	Effective during intervention
Autonomy	There is a choice not to comply	There is a choice not to comply	Compliance is compulsory
Consequences of behavior inconsistent with policy-driven change	Legally none, potentially social or economical	Legally none, potentially social or economical	Sanction or lack of permission to do something
Transparency	Not necessarily transparent	Necessarily transparent	Necessarily transparent and publicly available

Many of the differences and similarities between the approaches are due to the form in which each instrument appears. For the purposes of this paper, we can simply note the most common form in which a nudge is used, i.e., a stimulus implemented in a decision situation. The form in which boosts are used, on the other hand, most often involves the acquisition of specific competencies, e.g., as a result of risk perception training. Legal instruments, in contrast, usually take the form of regulations and prescriptions. In addition, it is also important to consider what the target audience of each intervention or instrument is. While the target audiences of nudges and boosts can be populations or selected individuals, the target audiences of legal tools are groups and populations. Furthermore, these interventions are usually implemented by different entities. Whereas nudges and boosts can be used or created by any sector (e.g., private, governmental, or non-governmental organizations), legal instruments can only be created by an authorized body, such as a legislature, which is usually a state authority.

Other major factors that should be taken into account when considering the implementation of a given tool are its cost, ease of implementation, and duration of action. Typically, nudges are described as the cheapest and fastest tools to implement. Implementation of boosts may be a bit more complicated because this intervention is aimed at the acquisition of competencies, which is not instantaneous. The implementation of legal tools is usually the most complicated because it involves legislative procedures that require substantial resources. In addition, relatively low or moderate implementation costs can directly result from the application of the proportionality rule, which sometimes may emphasize the use of behavioral tools. Behavioral interventions usually have a low cost of implementation; examples show that low-budget solutions that affect choice of architecture [12] are prevalent in social policy. The costs of interventions may also differ from the perspective of an individual, who is within a target audience. Nudges usually have the lowest individual cost because adjusting to defaults or social norms tends to occur automatically, without the need to engage in any deep cognitive processing of information [14]. However, individual costs increase in the case of boosts or legal regulations because they involve acquisition of competence or adjustment to legal prescriptions, which require additional effort and may sometimes be discouraging.

To understand the differences among these approaches, it is useful to look at them from the perspective of more physical criteria, such as duration. Duration is one of the dimensions in our list that distinguish nudges and boosts. Most often, the main effects triggered by a nudge are noticeable during the operation of the intervention. When removed, the desired policy-driven effects also disappear. Legal tools work in a similar way. The effects of boosts, on the other hand, can last long after the intervention has ended, e.g., if an individual learns a particular competency, such as compliance with COVID-19 regulations, those skills will stay with them for much longer.

Further distinguishing dimensions of these instruments are those related to autonomy and the potential consequences associated with behavior inconsistent with that defined by the tool. Typically, individuals may easily act contrary to the goals of behavioral interventions, but this is rarely the case for legal instruments. Similarly, the consequences of not behaving in accordance with the objective of the instruments differ; whereas there are no legal consequences of not complying with nudges and boosts, there might be sanctions or loss of permission to be able do something if one does not obey a particular regulation. If an individual does not choose to behave in the way advocated by behavioral interventions, the result is a lack of financial or social gain for doing so; however, in case of boosts or laws, there is an additional immediate gain of avoiding the effortful costs of acquiring new skills or obeying a law.

The last dimension in our list is the transparency of individual tools for citizens. Legal tools are published by the legislature and citizens can easily access them. This is not always the case for behavioral interventions – the question of whether awareness of the impact of behavioral tools affects their effectiveness is currently being widely debated [10]. However, most academics claim that while nudges are not necessarily transparent, boosts necessarily are. The effectiveness of boosts depends on whether the target audience fully understands the aim of competencies they can use [15]. In our view, the transparency criterion does not differentiate well between the two behavioral interventions we are discussing. Although nudges may have a covert, implicit character, and an individual may not be aware what choice is in line with a policy (which might violate autonomy), a similar problem may arise when boosts are used. Although boosts are transparent, there is no guarantee that the information provided to acquire a competency is objectively valid and serves a good purpose. However, such ethical concerns apply to all instruments designed to change behavior. In nudging situations, it simply might be harder to evaluate policymakers’ intentions.

To summarize, knowing the differences and similarities between these approaches can greatly facilitate their application by policymakers. It may turn out to be especially important in situations where resources, such as time, are limited, e.g., because the need to act is urgent – as faster behavioral interventions are quicker to implement than legal ones are. On the other hand, in situations where it is important that a behavior does not deviate from a rule, legal instruments might provide the best solution. The relationship between behavioral interventions and the law are not unidirectional, but rather mutual. We strongly believe that the law can support both nudges and boosts. In turn, nudges and boosts can support legal orders or prohibitions, and in such cases, behavioral interventions can have an additive effect, as in those situations where the use of masks is required to combat COVID-19, so that the law does not become a dead letter. Going further, boosts can be supported by the law by introducing minimal necessary qualifications. Then, each person could be required to develop a skill that aids decision-making in the long-term, even after the law is abolished.

### Behavioral interventions used to alleviate the effects of the COVID-19 pandemic

Behavioral interventions in the form of nudges and boosts can be employed to alleviate the effects of the COVID-19 pandemic in different areas [41]. A wide range of interventions have been adopted to promote the WHO recommendations: social distancing, wearing masks, and disinfection [42]. Apart from these, there are interventions to target socially unwanted behavior, a side effect of a pandemic – stockpiling behavior (also called hoarding) [43], chronic loneliness [44], and what might be called an “infodemic” [45]. The infodemic or massive spread of misinformation and disinformation about COVID-19 has become a threat to the efficacy of pandemic measures and the health of individuals [45]. Due to the different specifics of the interventions for the pandemic and the effects of the infodemic, these are considered separately below.

#### Interventions improving the efficacy of implemented measures and alleviating the pandemic’s effects

Behavioral interventions can be used to support government efforts to reduce the effects of the COVID-19 pandemic. Depending on the circumstances, nudges and boosts can be applied according to the specific situation, exploiting the design advantages of particular interventions. One such useful tool is the acronym DDM (Disinfection, Distance, Masks), a type of simple heuristic (a boost) proposed by the Polish Ministry of Health, which aims to equip citizens with a brief instruction, overcoming the uncertainty related to the current situation [46]. The actual effectiveness of the intervention is yet to be assessed, but the DDM phrase has been used by various media, providing a standard for citizens’ behavior. Furthermore, in some countries sanitation experts think the observed drop of 50% in the number of people falling ill from the flu, compared to the previous year, is a side-effect of introducing the DDM rule [47].

Decreasing individuals’ uncertainty can improve judgment or reduce unnecessary behavior. In many countries, hoarding behavior was identified as unwanted at the beginning of the pandemic in February and March 2020 [48]. Although it seemed an extraordinary phenomenon, hoarding has been observed before in cases of natural disasters, such as tornadoes [49], and it usually is explained as a way for individuals to regain a sense of control. An intervention targeting stockpiling behavior was proposed by Kim et al. [43]. The problem appears when consumption is excessive and individuals buy much more than they actually need. To address this problem, Kim and colleagues designed a nudge that provided participants with additional relative statistical information. In their study, researchers compared the threat caused by the coronavirus with the threats posed by car accidents or the flu. The results showed that presenting comparative statistics reduced the level of the perceived threat and stockpiling intentions [43].

Disinfection and overall hygiene is another area that is important from the perspective of strategies against COVID-19. The emergence of unwanted behavior has forced researchers and authorities to apply various solutions. In most countries, attempts to improve hygiene have involved hanging instructional posters adjacent to sanitation apparatus (e.g., sinks in public/office washrooms) to convince people to wash their hands more thoroughly [50]. Other methods can involve the implementation intention techniques based on the Gollwitzer [18] method to stop touching the face or “T-zone” (T stands for *touch*) [51] or by nudging people to use hand hygiene [52]. Recent findings show that hygiene can be effectively encouraged with either boosts or nudges [53]; however, this may not always be the case with nudges [52].

#### Interventions targeting the effects of the infodemic

According to the WHO [45], the coronavirus pandemic has led to an “*overabundance of information, both online and offline*,” which may have weakened attempts to decrease the effects of the pandemic in society. It is important to recognize that one can consider the use of both boosts and nudges in this area. The accuracy nudge [54] can substantially increase truth discernment in the sharing intentions of participants. Pennycook and colleagues used a simple accuracy reminder, which involved asking participants to assess the accuracy of particular headlines. The results showed that asking participants about their appraisal of the reliability of information was a much better strategy than asking them whether they wanted to share the information. When assessing one’s own sharing intentions, participants may be biased by the rewards given by social media platforms. Usually, instead of thinking about the trustworthiness or accuracy of particular information, we can be tempted by the instant gratification given by “likes” or other positive reactions [55]. Thanks to the accuracy nudge, it might be possible to switch an individual’s attention from immediate social reinforcements towards accuracy or reliability. From another perspective, the research emphasizes the lack of attention given by Internet users to information they encountered online – the more users attend to accuracy, the less likely they are to pass fake news on to others.

A fake news inoculation technique was also proposed in the context of COVID-19 [21]. According to the authors of the Bad News game, their practical intervention, based on the idea of active inoculation against fake news, can be conveniently transformed to work in the COVID-19 context. Furthermore, their original game has already been played by over 1 million people, which means it may not just be a tool for widespread dissemination, but also a means of creating a kind of “herd immunity” against the infodemic [21]. In the case of fake news “viruses,” it is not always important to immune the whole of society – it might be enough to make a sufficiently large number of people immune in order to break the chain of transmission.

#### Effectiveness of behavioral interventions during the COVID-19 pandemic

Limited findings indicate that the effectiveness of behavioral interventions may vary across tools, contexts, or individuals [4, 56]. It is important to look at the effects of behavioral interventions from the perspective of group differences. Many researchers and practitioners state there is nothing like a “one-size-fits-all” nudge and that general behavioral interventions should be personalized to work properly [15], and initial findings in the COVID-19 context confirm this approach. For example, Boruchowicz and colleagues [56] found that men showed more risky behavior than women, whereas older people were more likely to wear masks and to leave their homes less often than younger people. These findings were similar to those of Nivette et al. [4], which showed that Swiss men were also less likely to comply with new restrictions than women.

Among the various COVID-19-related problems that can be targeted by behavioral interventions, vaccination uptake has been the most promising so far. One approach that resulted in increased vaccination involved large-scale text messaging combined with classical nudges, such as defaults or reminders [57]. A large-scale randomized controlled trial by Dai showed that sending short text nudges that induced feelings of psychological ownership of the vaccine increased the vaccination rate by 26% [57]. Another approach was used by Rebitschek and colleagues [58], who implemented a boost intervention, i.e., simple fact boxes that presented comparisons of the benefits and harms of having a vaccination. The researchers reported that the intervention successfully increased vaccination knowledge and a positive evaluation of the risk connected to vaccination among sceptics [58].

Behaviorally informed messages may also be effectively used to tackle other issues emerging in the wake of pandemics. A study in Sao Paulo [56] showed that, compared to people who did not receive any message, individuals who received a text message referring to COVID-19 restrictions as a “civic duty” were over 12% more likely to report keeping an appropriate distance from others and 3% more likely always to wear masks. Successful use of messages to increase mask-wearing intentions were also reported by Capraro and Barcelo [59], when individuals were encouraged to “rely on their reasoning” instead of “relying on their emotions.” Another large-scale text-message study by Moriwaki et al. [60], which included 0.3 million users in Tokyo, found that nudges using loss-aversion effectively reduced the time individuals engaged in outdoor activities by approximately 52 min/weekend day. However, nudge-based messages do not always work, and any kind of behavioral intervention should not be treated as a universal panacea. For example, Bilancini et al. [61] reported no effect of social-norm nudges on people’s understanding of COVID-19 guidelines.

Although the recent rapid pace of research was extremely important for fighting the COVID-19 pandemic, there have been noticeable drawbacks [62]. One of these is the blurring of the definitions of nudges, boosts, and other interventions within the behavioral sciences. Blurring led to a situation in which researchers wrongly labeled the overall communication created by the Government of India as nudging [63]. Eventually, such misunderstandings can lead to losses in the actual effectiveness of interventions, which come in their unique forms and have distinctive key characteristics. It is important to emphasize that not all interventions that involve governmental communication are nudges. Some are simply public press releases, which can be an effective intervention, but clearly are not nudges, according to the classic literature [16]. By establishing a well studied and coherent framework, it is possible to obtain a firm basis for effective public policy strategies. We also recognize the fact that the popularity of nudge theory can draw attention from different domains, leading to unnecessary misconceptions. Therefore, we believe it is crucial to rely on a comprehensive approach like that presented in this paper, based on established theories. Mixing nudging and boosting may effectively enrich the policy maker’s toolbox. However, it should be remembered that if the government decides to use a nudge or boost, it should always do so in accordance with the principle of proportionality, and it should therefore consider whether this measure is necessary and suitable for the protection of the common good in the form of public health.

## Conclusions and further directions

Nudges and boosts have been effective in cases of regular, continuous problems, and they may be of crucial assistance in the face of new challenges, such as the COVID-19 pandemic, especially in areas where effective action is needed. Such a behavioral approach presents a challenge for effective implementation; however, as indicated by the evidence, to date, it supports the prescriptive nature of the legal system. The key weakness in the behavioral science approach is that there is relatively little evidence that favors one intervention over another. We are aware of only three empirical studies that compared nudges and boosts directly [53, 64, 65]. Only by such a methodical approach can researchers obtain a theoretical map to support the empirical findings, thereby informing us when it is better to use one intervention or another, or when they may be used interchangeably. Furthermore, for more complex systems and environments, it might also be possible to see additive or backfire effects in situations where both types of interventions are applied simultaneously**.** Exploration of synergy or exclusion effects must be the next avenue of research, which may result in multi-intervention programs exploiting the real potential of nudges and boosts.

The government’s application of a specific tool aimed at achieving public policy objectives requires the principle of proportionality. This is a basic condition for using both a specific tool (law, nudges, or boosts) and specific solutions within it (e.g., the choice of a specific standard in the case of law). It is possible, and in some cases it is also desirable, to use more than one tool (behavioral or legal). Given the need to shape health policy during the COVID-19 pandemic, where the state can regulate social life if it is interested in doing so, it can use any of the three tools. Therefore, it can use the traditional tool (i.e., law) or behavioral interventions. In Scenario I Row C (Fig. 1), nudges and boosts can support the law, and therefore the state can use traditional legal tools along with behavioral interventions in parallel to increase the chances of achieving its goals. Unlike Lepenies and Małecka [9], who proposed the use of nudging as one of two approaches to lawmaking (the law as an instrument, and the law as certain norms to be followed), we propose an approach that we believe is more transparent without reducing the power of the interventions. Furthermore, behavioral tools can support obedience to the law – legal norms that use behavioral interventions in their content. Despite resorting to the law as a traditional policy-making tool, the legislature can use the nudge construct as the content of the law. Nudges and boosts can also be independent tools for shaping public policy by the state. In Scenario II Row C, when the state cannot regulate a specific area in which it is interested, and therefore it cannot look to “the law,” nudges and boosts may serve as independent tools (see Fig. 1, Scenario II Row C).

We have demonstrated that an interdisciplinary approach that merges psychology and law adds nudges and boosts to the public policy toolbox, making legal regulations easier to follow and understand. The COVID-19 pandemic has highlighted the importance of rules that are transparent and easy to follow, and what can be achieved using behavioral interventions. The use of new or misunderstood restrictions in times of high uncertainty may lead to considerable non-compliance, risking the health or life many individuals [4, 5]. It is essential to use behavioral science to explain the law or public policy tools to lay people, especially during global crises such as pandemics. A strong partnership between public policy and behavioral science is the most effective way of leading societies through uncertain times. It is also important to acknowledge and be aware of the law and its boundaries. The most effective ways of combining the law and behavioral interventions have yet to be determined.

## Data Availability

Not applicable.

## Footnotes

1 Due to the fact that the subject of these considerations is related to public policy in the field of health implemented by the state, we refer to this concept as a systematic activity undertaken and implemented by public authorities, implemented within and on the basis of applicable laws, and aimed at solving problems with various areas of social life. For other definitions of policies undertaken by entities other than the state, see Howlett & Cashore [22]

## References

[1] Mahase E (2020). Covid-19: UK approves Pfizer and BioNTech vaccine with rollout due to start next week. BMJ..

[2] Worldometers. COVID-19 CORONAVIRUS PANDEMIC [Internet]. 2021 [cited 2021 Sep 11]. Available from: https://www.worldometers.info/coronavirus/

[3] WHO. Strengthening the Health System Response to Covid-19 Recommendations for the WHO European Region [Internet]. 2020 [cited 2021 Jan 4]. Available from: https://apps.who.int/iris/bitstream/handle/10665/333072/WHO-EURO-2020-806-40541-54465-eng.pdf?sequence=1&isAllowed=y.

[4] Nivette A, Ribeaud D, Murray A, Steinhoff A, Bechtiger L, Hepp U, Shanahan L, Eisner M (2021). Non-compliance with COVID-19-related public health measures among young adults in Switzerland: insights from a longitudinal cohort study. Soc Sci Med.

[5] Roy-Chowdury V, Perera D, Tagliaferri G, Mottershaw A, Egan M (2020). Young men are hardest to engage on coronavirus guidance [internet].

[6] Burris S, de Guia S, Gable L, Levin D, Parmet WE, Terry N (2020). Assessing legal responses to COVID-19. 2020 Assess Leg Responses to COVID-19 Bost Public Heal Law Watch.

[7] The White House Office of the Press Secretary (2015). Executive Order -- Using Behavioral Science Insights to Better Serve the American People [Internet].

[8] Halpern D. Inside the nudge unit: how small changes can make a big difference: Random House; 2015.

[9] Lepenies R, Małecka M (2015). The institutional consequences of nudging – nudges, politics, and the law. Rev Philos Psychol.

[10] Marchiori DR, Adriaanse MA, De Ridder DTD. Unresolved questions in nudging research: Putting the psychology back in nudging. Soc Personal Psychol Compass. 2017;11(1):e12297. 10.1111/spc3.12297.

[11] Thaler RH, Sunstein CR. Nudge: Improving Decisions about Health, Wealth, and Happiness: Yale University Press; 2008.

[12] Behavioural Insights Team (2015). The Behavioural Insights Team - Update Report 2013–15 [Internet].

[13] Sunstein CR (2015). Nudges do not undermine human agency. J Consum Policy.

[14] Grüne-Yanoff T, Hertwig R. Nudge Versus Boost: How Coherent are Policy and Theory? Mind Mach. 2016;26:149–83. 10.1007/s11023-015-9367-9.

[15] Hertwig R, Grüne-Yanoff T (2017). Nudging and boosting: steering or empowering good decisions. Perspect Psychol Sci.

[16] Sunstein CR (2014). Nudging: a very short guide. J Consum Policy.

[17] Hummel D, Maedche A (2019). How effective is nudging? A quantitative review on the effect sizes and limits of empirical nudging studies. J Behav Exp Econ.

[18] Gollwitzer PM (1999). Implementation intentions: strong effects of simple plans. Am Psychol.

[19] Kozyreva A, Lewandowsky S, Hertwig R (2020). Citizens versus the internet: confronting digital challenges with cognitive tools. Psychol Sci Public Interest.

[20] Roozenbeek J, van der Linden S (2019). Fake news game confers psychological resistance against online misinformation. Palgrave Commun.

[21] van der Linden S, Roozenbeek J, Compton J. Inoculating Against Fake News About COVID-19. Front Psychol. 2020;11:2928. 10.3389/fpsyg.2020.566790 PMC7644779 33192844

[22] Howlett M, Cashore B. Conceptualizing public policy. In: Comparative policy studies: Springer; 2014. p. 17–33.

[23] Schmitt NM, Schmitt J, Encycl Public Heal Kirch, W (2008). Definition of public health.

[24] Agénor P-R. Public capital, growth and welfare: analytical foundations for public policy: Princeton University Press; 2013. 10.23943/princeton/9780691155807.001.0001.

[25] WHO. Health policy [Internet]. [cited 2021 Jan 4]. Available from: https://www.euro.who.int/en/health-topics/health-policy/health-policy

[26] WHO (2016). Strategizing national health in the 21st century: a handbook [Internet].

[27] Gill M (2002). Building effective approaches to governance [internet].

[28] Birgand G, Castro-Sánchez E, Hansen S, Gastmeier P, Lucet J-C, Ferlie E, Holmes A, Ahmad R (2018). Comparison of governance approaches for the control of antimicrobial resistance: analysis of three European countries. Antimicrob Resist Infect Control.

[29] Resnik DB (2007). Responsibility for health: personal, social, and environmental. J Med Ethics.

[30] Bartoň M, Mates P. Public versus private interest–can the boundaries be legally defined? Czech Yearb Int Law. 2011:172–89. Available at SSRN: https://ssrn.com/abstract=1799331.

[31] Broniowski v. Poland (2004). European Court of Human Rights, no. 31443/96, § 149 [Internet].

[32] Jahn and others v. Germany (2004). European Court of Human Rights 46720/99, 72203/01 and 72552/01, § 80. European Court of Human Rights.

[33] European Parliament, European Council (2006). Directive 2006/123/EC of 12 December 2006 on services in the internal market.

[34] Bosphorus Airways v. Ireland (2005). European Court of Human Rights, Judgment of 30 June 2005, no 45036/98, § 150 [Internet].

[35] European Parliament, European Council. Charter of Fundamental Rights of the European Union, art. 52, sec. 1 [Internet]. 2012. Available from: https://eur-lex.europa.eu/legal-content/EN/TXT/?uri=CELEX%3A12012P%2FTXT

[36] Tridimas T (1999). Proportionality in European community law: searching for the appropriate standard of scrutiny. na.

[37] Polish Constitutional Tribunal (1998). Judgement no. 7, pos. 113, K 42/97.

[38] Polish Constitutional Tribunal (2000). Judgment no. 1, pos. 3, P 11/98.

[39] Alemanno A, Sibony A-L (2015). Nudge and the Law: A European Perspective by Alberto Alemanno and Anne-Lise Sibony (Eds) Oxford: Hart Publishing, 2015. Eur J Risk Regul.

[40] Van Aaken A. Constitutional limits to paternalistic Nudging: A proportionality Assessment. Forthcoming in: Alexandra Kemmerer, Christoph Möllers, Maximilian Steinbeis, Gerhard Wagner (eds.), Choice Architecture in Democracies, Exploring the Legitimacy of Nudging (Oxford/Baden-Baden: Hart and Nomos, 2015). 2015.

[41] Van Bavel JJ, Baicker K, Boggio PS, Capraro V, Cichocka A, Cikara M, et al. Using social and behavioural science to support COVID-19 pandemic response. Nat Hum Behav. 2020;4:460–71. 10.1038/s41562-020-0884-z. 10.1038/s41562-020-0884-z 32355299

[42] Michalek G, Schwarze R (2020). The strategic use of nudging and behavioural approaches in public health policy during the coronavirus crisis.

[43] Kim J, Giroux M, Gonzalez-Jimenez H, Jang S, Kim S (Sam), Park J, et al. Nudging to Reduce the Perceived Threat of Coronavirus and Stockpiling Intention J Advert 2020 29;1–15.

[44] Nault KA, Rogers BA, Sezer O, Klein N (2020). Behavioral insights for minimizing loneliness during the COVID-19 pandemic.

[45] WHO. Managing the COVID-19 infodemic: Promoting healthy behaviours and mitigating the harm from misinformation and disinformation [Internet]: World Health Organization; 2020. [cited 2021 Jan 4]. p. 9–11. Available from: https://www.who.int/news/item/23-09-2020-managing-the-covid-19-infodemic-promoting-healthy-behaviours-and-mitigating-the-harm-from-misinformation-and-disinformation%0Ahttps://www.who.int/news-room/detail/23-09-2020-managing-the-covid-19-infodemic-promotin

[46] Polish Ministry of Health (2020). Zero tolerancji dla nieprzestrzegających zasad bezpieczeństwa [Internet].

[47] Godlewska I (2020). Koronawirus pogonił grypę? O połowę mniej zachorowań. Gazeta Wyborcza Trójmiasto [Internet].

[48] Baddeley M. Hoarding in the age of COVID-19. J Behav Econ Policy, Society for the Advancement of Behavioral Economics (SABE). 2020;4(S):69–75.

[49] Yoon J, Narasimhan R, Kim MK (2018). Retailer’s sourcing strategy under consumer stockpiling in anticipation of supply disruptions. Int J Prod Res.

[50] Behavioural Insights Team (2020). Testing the efficacy of coronavirus messaging [Internet].

[51] Hallsworth M (2020). Behavioural Insights Team. How to stop touching our faces in the wake of the Coronavirus [Internet].

[52] Weijers RJ, de Koning BB (2020). Nudging to increase hand hygiene during the COVID-19 pandemic: a field experiment. Can J Behav Sci / Rev Can des Sci du Comport.

[53] van Roekel H, Reinhard J, Grimmelikhuijsen S (2021). Improving hand hygiene in hospitals: comparing the effect of a nudge and a boost on protocol compliance. Behav Public Policy.

[54] Pennycook G, McPhetres J, Zhang Y, Lu JG, Rand DG (2020). Fighting COVID-19 misinformation on social media: experimental evidence for a scalable accuracy-nudge intervention. Psychol Sci.

[55] Brady WJ, Crockett MJ, Van Bavel JJ (2020). The MAD model of moral contagion: the role of motivation, attention, and Design in the Spread of moralized content online. Perspect Psychol Sci.

[56] Boruchowicz C, Bóo FL, Pfeifer FF, Russo GA, Pacheco TS (2020). Are Behaviorally Informed Text Messages Effective in Promoting Compliance with COVID-19 Preventive Measures?: Evidence from an RCT in the City of São Paulo.

[57] Dai H, Saccardo S, Han MA, Roh L, Raja N, Vangala S (2021). Behavioral nudges increase COVID-19 vaccinations: Two randomized controlled trials. medRxiv.

[58] Rebitschek FG, Ellermann C, Jenny MA, Siegel NA, Spinner C, Wagner GG (2021). How skeptics could be convinced (not persuaded) to get vaccinated against COVID-19.

[59] Capraro V, Barcelo H (2021). Telling people to “rely on their reasoning” increases intentions to wear a face covering to slow down COVID-19 transmission. Appl Cogn Psychol [Internet].

[60] Moriwaki D, Harada S, Schneider J, Hoshino T. Nudging preventive behaviors in COVID-19 crisis: a large scale RCT using smartphone advertising: Institute for Economics Studies, Keio University; 2020.

[61] Bilancini E, Boncinelli L, Capraro V, Celadin T, Di Paolo R. The effect of norm-based messages on reading and understanding COVID-19 pandemic response governmental rules. arXiv:200503998 [physics] [Internet]. 2020 [cited 2021 Nov 6]. Available from: http://arxiv.org/abs/2005.03998.

[62] Dinis-Oliveira RJ (2020). COVID-19 research: pandemic *versus* “paperdemic”, integrity, values and risks of the “speed science”. Forensic Sci Res.

[63] Debnath R, Bardhan R. India nudges to contain COVID-19 pandemic: A reactive public policy analysis using machine-learning based topic modelling. PLOS ONE. 2020:15(9):e0238972. 10.1371/journal.pone.0238972 PMC7485898 32915899

[64] Bradt J. Comparing the effects of behaviorally informed interventions on flood insurance demand: an experimental analysis of ‘boosts’ and ‘nudges’. Behav Public Policy. 2019:1–31. 10.1017/bpp.2019.31.

[65] Franklin M, Folke T, Ruggeri K (2019). Optimising nudges and boosts for financial decisions under uncertainty. Palgrave Commun.

